# A Novel Electromagnetic-Neurobiologic Interface for Functional Animation of Dormant Motor Nerve Roots in Spinal Cord Injury via Neuromodulation

**DOI:** 10.3389/fsurg.2019.00073

**Published:** 2020-01-10

**Authors:** Jawad Shah, Richard H. Smith, Abeer Al-Gharaibeh

**Affiliations:** ^1^Department of Neurosurgery, Insight Institute of Neurosurgery & Neuroscience, Flint, MI, United States; ^2^Department of Research, Insight Institute of Neurosurgery & Neuroscience, Flint, MI, United States; ^3^Center for Cognition and Neuroethics, University of Michigan-Flint – IINN, Flint, MI, United States; ^4^Department of Medicine, College of Human Medicine, Michigan State University, East Lansing, MI, United States

**Keywords:** spinal cord injury, paralysis, electrical stimulation, neuromodulation, interface

## Abstract

Complete spinal cord injury is a devastating occurrence afflicting millions of people worldwide with no available treatment for functional motor recovery. In this report, we describe a procedure in which we used parts of a device available for chronic pain treatment to provide a neuromodulation of motor nerve roots in a case with complete motor and sensory paraplegia. By using a retrograde trans-foraminal approach to implant electrodes close to L2-S1 motor nerve roots bilaterally, we were able to stimulate those nerves and induce precise movements at the joints of lower extremity in a T5 complete spinal cord injury case. We believe that our approach shows potential of the device as a rehabilitation system with the possibility of a parallel electric circuitry that can bridge a damaged spinal cord.

## Introduction

The estimated annual incidence of spinal cord injury (SCI) is ~54 cases per million population in the Unites States, with about 17,000 new SCI cases each year (1). Many studies have focused on standing and walking restoration in spinal cord injury (2–5). Stem cells and pharmacologic agents have shown potential, but no recovery of motor functions was achieved (6–8). However, neuromodulation using electrical stimulation has proven effective (3–5). It is hypothesized that this neuromodulation is taking advantage of the remaining intact neural circuitry below the level of the injury site (4, 9, 10). In many SCI cases, those remaining neural pathways are disconnected physiologically or anatomically from proximal central neurons limiting transmission to and from the cerebral cortex (9–11).

There are three main approaches for spinal cord stimulation; epidural, transcutaneous, and intraspinal (10, 12). In recent decades, several pre-clinical and clinical studies have indicated that electrical epidural stimulation (EES) have a potential effect in SCI treatment (13–20). Recently, two groups showed that EES accompanied with intensive physical rehabilitation enabled patients with incomplete SCI—where there is partial preservation of motor and sensory functions or complete loss of motor functions with preservation of some sensory functions—to stand and walk again (3, 5). However, the lack of efficacy in complete paralysis cases, where there is complete severing of spinal cord, makes it necessary to find alternative treatments strategies. Studies that involve stimulation directly on the motor nerves by implanting electrodes within the spinal cord are rare, but results from pre-clinical studies provide insights into the potential benefits of this approach (21, 22).

We hypothesize that using parts of the devices available for the treatment of chronic pain to target dormant motor neurons through trans-foraminal approach and directly implant electrodes near nerve roots will lead to precise functional movements in complete SCI patients. To the best of our knowledge, this report describes a first attempt at inducing locomotor function by direct electrical stimulation of motor nerve roots using retrograde advancement of leads with trans-foraminal approach in complete SCI patient.

## Case Presentation

### History and Examination

A 26-year old woman presented with traumatic complete T5 paralysis for 2 years after suffering a T5 burst fracture caused by falling from a tree while hunting. T5 posterior fusion and decompression was performed to stabilize her, but the injury was complete with no return of function after 2 years. A baclofen pump was inserted into the L3 level for spasticity after few months of the injury. The severity of the injury was classified as grade A using the American Spinal Injury Association (ASIA) grading, which is defined as complete loss of motor and sensory functions below the level of the injury (23).

### Surgery

**Figure 1:**
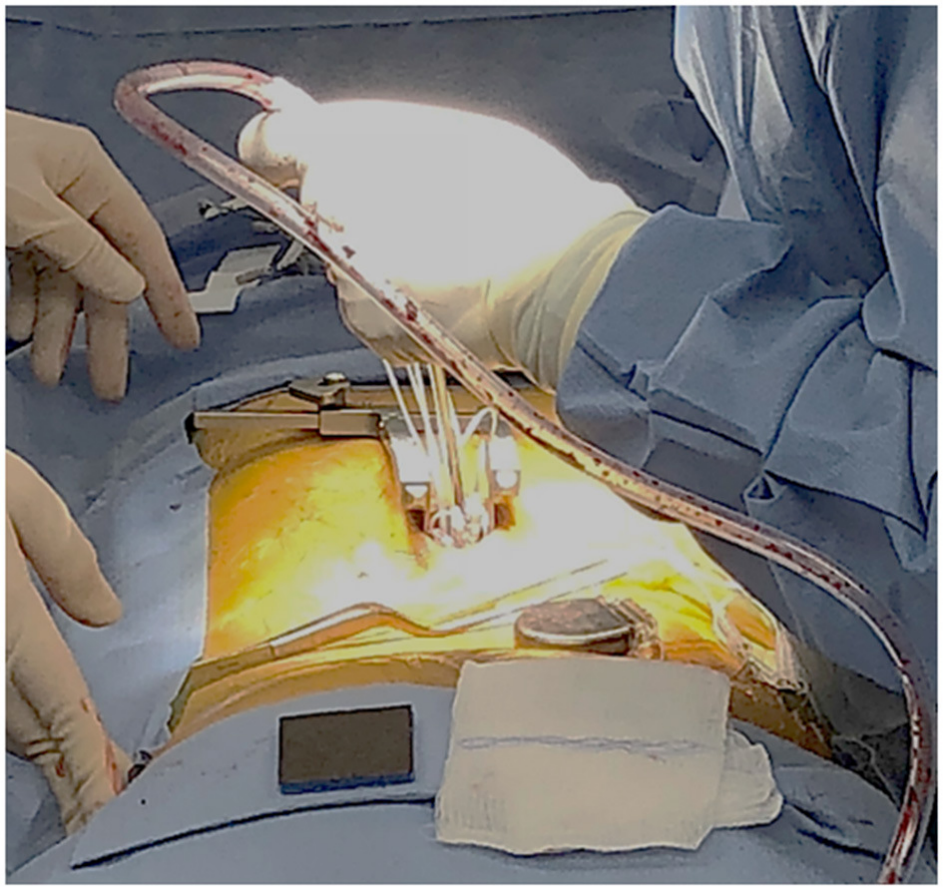
The dorsal antero-posterior incision site for the laminotomy at T12 provided access of about 2 cm per side. Stimulator leads, each having an 8-electrode array, were guided and implanted under fluoroscopy.

**Figure 2:**
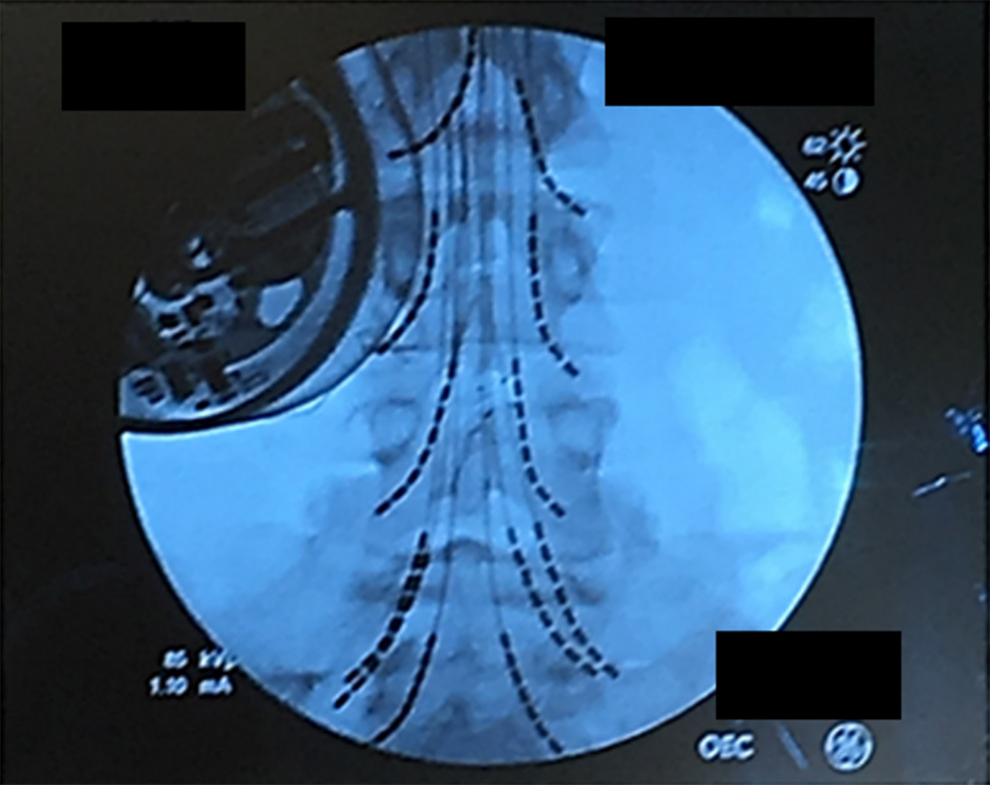
Lead placement showing the final position of the 12-lead array under fluoroscopy. Leads were individually advanced to allow activation of spinal nerve roots from L2 to S1. The patient's baclofen pump is on the upper right.

The stimulator that was used in this study is approved by FDA for chronic pain treatment (24). After obtaining informed consent from the patient, a total of 8 leads with 8 electrodes each were implanted at L2, L3, L4, and S1 bilaterally. Four leads for a total of 16 electrodes on each nerve root with two leads each were implanted at L5 bilaterally. The procedure was performed under general anesthetic with a small laminotomy at T12 to facilitate retrograde advancement and insertion of percutaneous leads to each level (Figure 1). The procedure was done and guided under fluoroscopy and the total surgical time was ~120 min. A total of 2 batteries, with four ports each, were utilized connecting in the following configuration: 8 electrodes to D port for S1, 16 electrodes with a splitter to C port, 8 electrodes from L4 and 8 electrodes to L3 with a splitter to B port, and 8 electrodes to A port from L2. This allowed activating every other contact on each lead that is plugged in the splitter. Electrodes were then anchored into the interspinous ligament and tunneled to two separate gluteal pockets on either side. All electrodes from the left were sent to the left battery and right electrodes to the right battery. This procedure allowed for a total of 64 contact points, 32 per battery, on 12 leads with implantation of 96 electrodes that covered L2 to S1 bilaterally. Each lead hugged the posterolateral gutter of the spinal canal before exiting along with the nerve root out the respective foramen (Figure 2).

### Post-surgical Course

After recovery, the patient was sent home the same day, and allowed 1 week for healing. She was then brought to an outpatient setting. The device was programmed to send electrical signals through selected implanted electrodes. Those programs were numbered and linked to the movement of different muscle groups which can be controlled by the patient using a remote control. On the first day of using electrical stimulation, the stimulator was shown to raise her leg with full leg bending and extension movements (Video S1). Increases in muscle strength with stimuli were measured and results are shown in Table 1. Patient was also able to stand with stimuli and a weight-bearing device (Rifton Tram Lift) for a maximum of 54 s before fatigue in 60-min sessions with 8 trials.

**Table 1:** Lower extremity Muscle groups strength^*^.

**Muscle groups**	**Strength of muscle groups/with electrical stimulation**	**Strength of muscle groups/without electrical stimulation**
Right ankle dorsiflexors	4	0
Left ankle dorsiflexors	4–	0
Right knee extensors	4	0
Left knee extensors	4	0
Right knee flexors	4	0
Left knee flexors	4	0
Right Hip flexors	3–	0
Left hip flexors	3–	0
Right hip abductors	4–	0
Left hip abductors	4–	0

* *Lower extremity motor score: (0/5): No contraction; (1/5): Muscle flicker, but no movement; (2/5): Movement is possible, but not against gravity; (3-/5): Gradual release from test position. (3/5): Holds test position against gravity, but not against resistance by the examiner; (3+/5): Holds test position against slight resistance; (4–/5): Holds test position against slight to moderate pressure; (4/5): Holds test position against moderate resistance; (4+/5): Holds test position against moderate to strong pressure; (5/5): normal strength; Holds test position against maximal resistance*.

## Discussion

This case report demonstrates the ability to modulate motor nerve roots in precise, replicable, and targeted manners in a patient who was diagnosed with complete sensory and motor (ASIA- grade A) SCI. The stimulator in this study uses externally provided electrical fields, placed upon or implanted within clusters of nerves including nerve roots. This case study suggests that parts of devices for treatment of chronic pain may be capable of causing muscle contraction among paralyzed patients. Although activation of the sensory roots cannot be excluded, the smoothness, speed, and precision of movements suggest that they result from the direct motor roots activation. However, further analysis using objective models of EMG may help in differentiation.

While minimal invasive insertion methods could be utilized for such a surgery, we started with an open approach to target the correct motor pool levels. In this case, the anatomy of the interlaminar space would make it difficult for an appropriate trajectory of insertion without a laminotomy for retrograde insertion of leads. While we think a laminotomy may be avoided by using extra-foraminal approach to each nerve root, antegrade placement bends on introducers wires, or in patients with large interlaminar space anatomy, the approach in this case was taken to minimize surgical time and achieve higher accuracy of electrodes placement. When electrodes are fully implanted in close proximity to the nerve, more precise targeting can be achieved using smaller current densities which are less likely to damage the cells (21). In this case, we also used multiple electrodes which we think will decrease the fatigability and increase the power and length of muscle contractions. Multi-contact cuffs or multiple independent nerve-based electrodes can delay the fatigue by alternating activation of independent motor unit pools within a muscle (25). To allow more programmability options without significant increase in the procedure cost, we used only 2 generators, but we added 2 leads to L5, and used multiple splitters. It is important to mention that using more generators and a larger array of leads for all targeted nerve roots in similar patients would be more effective and could help in specific task and fine control movements.

Previous studies on EES showed that there is a need for intensive physical therapy program following electrical stimulation to have a significant change (3–5). Similarly, we think that this strategy when combined with rehabilitation toward improving volitional muscle control could lead to improved function and independence. Electrical stimulation should be combined with extensive physical rehabilitation to activate motor pathways and strengthening synapses (10), as has been shown in many epidural electrical stimulation studies (3–5). Future plans for the patient include regular physical therapy sessions with focusing on increasing muscle bulk and creating specific-task movements.

This approach shows the potential use of the device as a rehabilitation procedure in complete SCI. Future directions will focus on optimal stimulation parameters, where precise targeting of the nerve roots could lead to more diverse functional effects. There are other major challenges might affect the efficacy of the procedure which need to be further tested. High electrical intensity might lead to cell damage while low intensity might not recruit contraction force enough for movement. Although we implanted the electrodes close to the motor nerve roots which decreases the need for high electrical intensities to achieve movements, the required electrical intensity to cause muscle contractions might vary among individuals or depending on their positions, in addition, paralyzed muscles in SCI patients fatigue excessively. Ambulation will require better lead design, sequencing programs, and weight carrying devices to allow a safe attempt at gradual return to walking. Developing algorithms to define movements that require overlapping activation of muscle groups, and stimulation protocols that preserve proprioceptive information will be needed to achieve stepping. Also, while multiple contacts offer a wide programmability options, developing a cuff lead with circumferential electrodes involving the smallest possible contact points might be capable of generating a greater variety of electron clouds and allow greater possibilities of stimulation.

We think that this approach, if coupled with precise computer algorithms and accompanied with rehabilitation programs, holds promise for a functional recovery (including ambulation) in complete SCI patients. An entire feedback loop might be activated when a signal that is gleaned from motor stimulation can be generated to send precise signals to a neuromodulator above the level of the injury. We also think that the full feedback loop would become closed if information can be harvested from the motor cortex and delivered to the motor nerve through the stimulation device. This could lead to a full parallel electrical artificial spinal cord that would bridge the injury site.

We believe that the current case shows the possibility of a parallel electric circuitry that can effectively bridge a damaged spinal cord. The current system provides benefit to the patient including independent ability to stand as well as exercise of muscle groups. As resources, time, and expertise are put into developing the current techniques to the next level, a new era of mobility for paralyzed patients may be ushered in.

## Data Availability Statement

The datasets are available upon request from the corresponding author.

## Ethics Statement

Ethical review and approval was not required for the study on human participants in accordance with the local legislation and institutional requirements. The patients/participants provided their written informed consent to participate in this study. Written informed consent was obtained from the individual(s) for the publication of any potentially identifiable images or data included in this article.

## Author Contributions

JS: conception and design, surgery, analysis and interpretation of data, and manuscript writing. RS: analysis and interpretation of data and manuscript writing. A-AG: analysis and interpretation of data, manuscript writing, and administrative support. JS, RS, and A-AG: final approval of the manuscript.

### Conflict of Interest

The authors declare that the research was conducted in the absence of any commercial or financial relationships that could be construed as a potential conflict of interest.

## Supplementary Material

Video S1 Externally applied electrical potential directed through the implanted spinal cord electrodes causes muscle contraction, raising the patient's leg. The ability of electrical stimulation to cause muscular contraction indicates that the primary insult is within nerve transmission and is not due to muscular weakness. A priori, the patient's task is to regain nervous control of muscle contraction.
